# *In vitro* utilization of lime treated olive cake as a component of complete feed for small ruminants

**DOI:** 10.14202/vetworld.2015.109-115

**Published:** 2015-01-29

**Authors:** A. Ishfaq, R. K. Sharma, A. Rastogi, B. A. Malla, J. Farooq

**Affiliations:** 1 Division of Animal Nutrition, Faculty of Veterinary Science, Sher-e-Kashmir University of Agricultural Sciences & Technology of Jammu, Jammu and Kashmir, India; 2 Department of Animal Nutrition, National Dairy Research Institute, Karnal, India

**Keywords:** acid detergent insoluble nitrogen-non, lime, male goats, olive cake, truly degradable organic matter

## Abstract

**Aim:**

The current *in vitro* study was carried out to determine the chemical composition and inclusion level of lime treated olive cake on acid detergent fiber (ADF) replacement basis in adult male goats.

**Materials and Methods:**

Crude olive cake was collected and evaluated for proximate composition and protein fractionation. It was treated with 6% lime and incubated for 1 week under room temperature in 2 kg sealed polythene bags and was evaluated for proximate composition after incubation. Different isonitrogenous complete diets containing 0-50% of lime treated olive cake on ADF replacement basis were formulated as per the requirement of adult male goats. In ADF replacement, fiber and concentrate sources were replaced by lime treated olive cake by replacing the 0-50% ADF percentage of the total 40% ADF value of complete feed. The formulated complete diets were tested for *in vitro* degradation parameters.

**Results:**

Treatment of olive cake with 6% slaked lime increased availability of cellulose and alleviated digestibility depression caused by high ether extract percentage. Organic matter, nitrogen free extract, ADF and neutral detergent fiber were significantly lowered by lime treatment of olive cake. The cornell net carbohydrate and protein system analysis showed that non-degradable protein represented by acid detergent insoluble nitrogen (ADIN) was 21.71% whereas the non-available protein represented by neutral detergent insoluble nitrogen (NDIN) was 38.86% in crude olive cake. The *in vitro* dry matter degradation (IVDMD) values were comparable at all replacement levels. However, a point of inflection was observed at 40% ADF replacement level, which was supported by truly degradable organic matter (TDOM), microbial biomass production (MBP), efficiency of MBP and partitioning factor values (PF).

**Conclusion:**

In our study, we concluded that there is comparable difference in composition of Indian olive cake when compared with European olive cake. The most important finding was that about 78% of nitrogen present in Indian olive cake is available to animal in contrary to that of European olive cake. We concluded from *in vitro* studies that Indian olive cake can be included in complete feed at 30% level (w/w; 40% ADF replacement) for feeding in small ruminants without compromising *in vitro* degradability of the feed.

## Introduction

The shortage and increasing cost of conventional feed ingredients have driven the attention of research towards utilization of non-conventional feedstuffs in livestock ration. The use of non-conventional feedstuffs minimizes the competition of livestock with humans for conventional food grains and reduces the cost of animal production [1-3].

Olive (*Olea europaea* L.) oil industry by-products are promising unconventional feedstuffs [4-6]. The introduction of olive cultivation coincides with the expansion of the Mediterranean civilizations [7]. Originating in Asia Minor, the olive tree spread beyond the Mediterranean (e.g. Southern Africa, Australia, Japan, China, United States of America).

Both cultivation of olive trees and olive oil extraction generate substantial amounts of by-products, which are potential pollutants [8]. However, they may be utilized in ruminant ration reducing their pollution potential as well as providing non-conventional feedstuff. In India, many states are taking keen interest in olive production. The integration of this olive cake in ruminant ration is limited. The main limiting factors are low crude protein (CP); relatively high fat content; high fiber and lignin content all contributing to its poor digestibility. Depending upon the processing, CP in olive cake varies between 8% and 12% dry matter (DM), but almost 80-90% of nitrogen is fixed on lignocellulose, thus not digestible [9]. The different oil extraction procedures and resulting by-products have been documented by Alburquerque *et al*. [10]. Olive cake is characterized by high variability of residual water (25-30%) in relation to extraction method; high percentage of crude fiber (27-41%) [11], particularly rich in lignin (28.9%); low CP (7.26%) [12] but a surprisingly high oil percentage (10.15%) [6]. Further, researchers have shown its poor digestive utilization in ruminants. This may be attributed to decrease in activity of the rumen microflora by 40% after ingestion of crude olive cake [13].

Various theories have been advanced to explain the reason for poor digestibility. High fat content, its composition and lignocellulosic nature of olive cake have been suggested as the incriminating factors. Buysse [14] has shown that ruminants are sensitive to intake of fat above 5% of DM in the ration. Further, Nefzaoui [12] suggested that there is the same phenomenon of “protection” of carbohydrates related to lignin with olive cake as occurs with straw and when olive cake was treated with alkalis, it’s *in vitro* digestibility increased by almost four times. In another study at author’s laboratory, Ashraf [5] suggested that treatment with slaked lime (Ca[OH]_2_) can help in addressing both the factors that limit the utilization of olive meal. In principle, lime can help in weakening lingo-cellulosic structure of olive meal, as well as it can form calcium salts with the free fatty acids of olive, thereby alleviating their depressing action on digestibility and still maintaining their availability to the animal. Bashir [6] reported that untreated olive cake can replace 7.5% of concentrate mixture (25% maize replacement) in goat ration without affecting digestibility. However Ashraf [5] was able to replace 12% of concentrate mixture (40% maize replacement) after treatment with slaked lime.

Olive cake is a concentrate cum roughage feedstuff as it has high energy content as well as high fiber content. It was hypothesized that utilization of olive cake can be increased in the ration by incorporating lime treated olive cake in complete feed, where its high fiber content can act as roughage and high fat content can act as a replacer of carbonaceous concentrate feedstuffs.

The present *in vitro* study was carried to determine the chemical composition, and inclusion level of lime treated olive cake on acid detergent fiber (ADF) replacement basis for male adult goats.

## Materials and Methods

### Ethical approval

The experiment followed the guidelines of Institutional Animal Ethics Committee.

This study was carried in the laboratory of Division of Animal Nutrition, Shere Kashmir University of Agricultural Sciences and Technology-Jammu, India.

### Collection of olive cake

Olive cake was collected from olive oil extraction mill owned by Advanced Centre for Horticulture Development, Govindpura, Ramban, Department of Horticulture, Jammu and Kashmir Government, India. Olive cake was brought to the laboratory and sun-dried to reduce to constant moisture level for 3 days. It was finely ground in the Sonar grinder (Associated Scientific Technologies, Delhi) and sieved through a mesh (1 mm).

Ground olive cake was treated with 6% lime as per Ashraf [5] with little modification. Briefly slaked lime solution was prepared by dissolving 6 g of slaked lime in 20 ml distilled water and was sprinkled over 100 g of olive cake spread out on a polythene sheet. The samples were dried in sunlight for 4 h and packed in sealed polythene bags for a week’s incubation in the laboratory.

### Proximate analysis and fiber fractionation

Proximate analysis of olive cake sample was performed as per AOAC [15] and fiber fractions were analyzed as per the method of Van Soest *et al* [16].

### Protein fractionation

Protein fractionation was done as per cornell net carbohydrate and protein (CNCP) system by Sniffen *et al*. [17]. The protein fractions are estimated by determination of feed nitrogen solubility in borate phosphate buffer in conjunction with analysis of acid detergent insoluble nitrogen (ADIN) and neutral detergent insoluble nitrogen (NDIN).

### *In vitro* gas production

Different isonitrogenous complete diets containing variable levels of lime treated olive cake on ADF replacement basis were formulated as shown in Table-1 as per the nutrient requirement of adult male goats by Ranjhan standard [18]. The formulated complete diets were tested for *in vitro* degradation parameters as per Menke and Steingass [19].

**Table-1:** Inclusion levels of lime treated Indian olive cake on ADF replacement levels*

Levels	L0	L1	L2	L3	L4	L5	L6	L7	L8	L9
Ingredient	0%	10%	15%	20%	25%	30%	35%	40%	45%	50%
Maize	10	9.25	8.5	7.75	7	6.25	5.5	4.75	4	3.25
Wheat bran	10	9.25	8.5	7.75	7	6.25	5.5	4.75	4	3.25
Mustard oil cake	13	13	13.1	13.3	13.5	13.7	13.8	14	14.1	14.25
Olive cake	0	7	11	15	18.3	22.5	26.7	30.5	35.45	39.9
Wheat straw	66	60.5	57.9	55.2	53.2	50.3	47.5	45	41.45	38.35

* The lime treated olive cake replaces all ingredients of feed by keeping uniform ADF content (40%) of each feed level *viz*. 7% olive cake in L1 replaces10% ADF of zero level and successively other levels replace corresponding percentage of ADF of zero level, ADF=Acid detergent fiber

#### Weighing of substrates and greasing of syringes

The samples were ground in Sonar grinder (Associated Scientific Technologies, Delhi) and passed through 1.0 mm screen and stored in air tight containers for use. Weighing was done on a weighing boat with removable stem so that the sample was put at the bottom of the syringe without leaving sample sticking on its side. The samples in the required proportion (of different inclusion levels) were introduced at the bottom of syringes. After weighing of samples, the piston was greased with white petroleum jelly (M.P. 53-57°C) and pushed into the barrel of the syringe. Lubrication with Vaseline makes syringes gas/water tight and prevents the piston from getting stuck in the barrel.

#### Preparation of buffer media

All the solutions were prepared as per Menke and Steingass [19]. All the solutions were prepared well before the start of incubation except the reduction solution, which was prepared freshly each time shortly before the rumen fluid was collected. The solutions were poured into a Woulff flask, mixed with a magnetic stirrer and warmed to 39°C in a water bath with digital thermostat as per the composition shown in Table-2. Carbon dioxide gas was passed through the submerged tube in the Woulff flask continuously during buffer media preparation.

**Table-2:** Composition of *in vitro* solution

Artificial saliva	Volume (ml)
Final volume	1000
Distilled water	475.0
Macromineral solution	240.0
Buffer solution	240.0
Micromineral solution	0.12
Resazurin	1.22
Reducing solution	
Distilled water	47.5
1M NaOH	2.0
Na_2_S_9_.H_2_0(mg)	336

On the day of incubation, the mixture of rumen liquor and particulate matter (*approximately* 60:40) was collected from local slaughter house into pre-warmed CO_2_ filled thermos and carried to the laboratory. The rumen fluid was bubbled with CO_2_ gas for few minutes and then mixed in a laboratory blender at medium speed to remove microbes attached to particulate matter. Rumen liquor was then strained through a double layer of muslin cloth. Strained liquor was then added to the buffer media when the media became colorless. Handling of rumen liquor was done under continuous flushing with CO_2_.

#### Filling of syringes and incubation

The buffered rumen fluid (30 ml) was dispensed to each syringe by a accurately marked self-made dispenser. After recording initial volume (± 0.5 ml), the syringes were placed in the incubator maintained at 39°C. The syringes were shaken by hand intermittently and once again after 6 and 12 h. If the gas volume exceeded 80 ml mark, gas was released and the incubation was continued. All incubations were run in triplicate and four syringes with buffered rumen fluid were incubated as blanks. A 0 h blank in duplicate was also kept during dispensing of buffered rumen fluid into syringes. At the end of incubation (24 h) the amount of gas produced was measured by reading the position of the plug and the contents of the syringes were analyzed further.

#### Determination of substrate degradation and microbial bio-mass production

The contents of the syringes were transferred to 500 ml spoutless beakers by repeated washings with neutral detergent solution without sodium sulfite. The contents were then refluxed for 1 h to extract the microbial matter from the undegraded feed, and the residue was recovered in pre-weighed filter crucibles. After drying the crucibles (with residue) at 105°C to constant weight, ashing was done at 400-500°C for 2 h. Truly degradable organic matter (TDOM), “microbial biomass production (MBP)”, “efficiency of MBP (EMP)” and “partitioning factor (PF)” were calculated as per Blummel *et al*.[20]:

TDOM = Feed (OM) incubated - residue (OM)

MBP = TDOM - (2.2 × net gas volume)

EMP = (TDOM – [2.2 × net gas volume]) × 100/TDOM

PF = TDOM/net gas volume

### Statistical analysis

Generalized linear model analysis of variance procedure [21] was used for results and the means having significant difference were ranked as per Duncan’s multiple range test [22].

## Results

### Proximate composition and fiber fractions of olive cake

The proximate composition and fiber fractions of olive cake and lime-treated olive cake used in this study are shown in Table-3. The OM (percent DM) in lime treated olive cake was found significantly (p<0.01) lower than crude olive cake. The ADF and NDF (Neutral Detergent Fiber) content was also found significantly (p<0.05) lower in lime treated olive cake. The acid insoluble ash and calcium was significantly higher in lime treated olive cake (p<0.05), whereas CP levels were comparable (p>0.05) in crude and lime-treated olive cake. The comparative composition of Indian olive cake and European olive cake is shown in Table-4.

**Table-3:** Percent chemical composition of Indian crude olive cake and lime treated olive cake

Attributes*	Crude olive cake	Lime treated olive cake	p value
Moisture	3.76±0.03	4.51±0.10	0.002
Organic matter	98.43±0.07	93.75±0.52	0.001
CP	5.83±0.73	6.57±0.17	0.379
EE	12.54±0.26	12.14±0.63	0.584
Total ash	1.57±0.07	6.25±0.52	0.001
NDF^a^	80.33±0.33	63.33±0.67	0.000
ADF^b^	62.00±0.58	54.67±0.33	0.000
Hemicellulose	18.33±0.88	8.67±0.88	0.001
Acid detergent lignin^c^	23.22±0.94	14.06±0.66	0.042
AIA	0.38±0.02	0.56±0.06	0.033
Calcium	0.26±0.01	4.46±0.43	0.001
Phosphorus	0.21±0.01	0.22±0.01	0.275

* All attributes expressed as percent DM except moisture,

a NDF-assayed without heat stable amylase and expressed exclusive of residual ash

b ADF-expressed inclusive of residual ash,

c Lignin (sa)-determined by solubilization of cellulose with sulfuric acid, NDF=Neutral detergent fiber, ADF=Acid detergent fiber, AIA=Acid insoluble ash, CP=Crude protein, EE=Ether extract

**Table-4:** Chemical composition (% DM) of Indian olive cake in comparison with European olive cake

Attributes	Indian olive*	Molina Alcaide and Yáňez Ruiz [43]**
Organic matter	97.56	90.1^a-e^
CP	6.61	7.26^a-e^
EE	11.11	5.45^b-e^
NDF	71.57	67.6^a-e^
ADF	56.66	54.4^a-e^
Acid detergent lignin	23.22	28.9^b-e^
Calcium	0.24	
Phosphorus	0.19	

All values are expressed as per cent DM except moisture

* Mean values observed at author’s laboratory by, Ashraf [5], Bashir[6] and author.

** World olive analysis;

a Al-Jassim *et al.* [13],

b Al-Masri [24],

c Al-Masri [31],

d Martín García *et al.* [26],

e Molina Alcaide *et al.* [35], DM=Dry matter, EE=Ether extract, CP=Crude protein, NDF=Neutral detergent fiber, ADF=Acid detergent fiber

### Nitrogen fractionation of crude olive cake

The ADIN (% total nitrogen) was 21.71% whereas the NDIN (% total nitrogen) was 38.86%. Around 78% of nitrogen is likely to be available to the ruminant. Fraction A which gets instantaneously solubilized in rumen and is cent percent digested in the intestine was 33.69%. The protein fractions likely to be degraded in the rumen were 56% and undegradable dietary nitrogen component was 22.29% (Table-5).

**Table-5:** Nitrogen fractionation of crude olive cake

Fractions	Percentage
A	33.69
B_1_	22.31
B_2_	05.14
B_3_	17.15
C	21.71
Neutral detergent insoluble nitrogen	
%DM	0.34
%NDF	0.42
%TN	38.86
Acid detergent insoluble nitrogen	
%DM	0.19
%ADF	0.30
%TN	21.71
Nitrogen solubility (%TN)	
Soluble total nitrogen	56.00
Soluble non-protein nitrogen	33.69
Soluble true protein nitrogen	22.31

NDF=Neutral detergent fiber, ADF=Acid detergent fiber

### *In vitro* DM degradability variables

The IVDMD values were comparable (p>0.05) between different inclusion levels. The IVDMD values varied from 48.75% to 56.25%. The TDOM (mg/200 mg DM) values varied from 97.50 to 112 with significant (p<0.01) difference among various replacement levels. The 40% ADF replacement level differed significantly (p<0.01) in TDOM from all other levels except 30% and 35% replacement level. The gas volume varied from 24 to 33 ml/200 mg DM and the levels differed significantly (p<0.01) from each other. The MBP value varied from 33.45 to 48.80 mg/200 mgDM. The MBP value was highest in 40% ADF replacement level and differed significantly (p<0.01) from all other levels. The EMP values varied from 33.42 to 46.41(%TDOM) and differed significantly (p<0.01) in various replacement levels. The values were highest in 40%, 45%, and 50% ADF replacement levels. The PF values varied from 3.31 to 4.12 and the values differed significantly (p<0.01) in various replacement levels. The values were highest and comparable (p>0.05) in 40%, 45% and 50% ADF replacement levels.

## Discussion

### Proximate composition and fiber fractions of crude and lime-treated olive cake

Proximate composition of the olive cake was similar to that reported by Ahmad [4], Ashraf [5], Bashir [6], Sansoucy [9] and Mioc *et al*. [23]. CP content (%DM) was in agreement to that provided by Al-Jassim *et al*. [13], Al-Masri [24], Vargas-Bello-Pérez *et al* [25], Martín García *et al*. [26], Molina-Alcaide *et al*. [27] and Sadeghi *et al*. [28]; but were higher than the values of Chiofalo *et al*. [29] and Gul *et al*. [30]. The percent ether extract (EE) content was higher than that reported by Al-Masri [24], Al-Masri [31], but is in agreement to the values of Ashraf [5], Bashir [6], Alhamad *et al*. [32], Luciano *et al* [33], Awawdeh and Obeidat [34] and Vargas-Bello-Pérez *et al* [25].

Chemical composition of olive cake has been shown to be influenced by factors such as geographical origin, procedure of production and processing [27]. Differences in terms of CP and EE content of the olive cake between some of the previous reports and results of this study may be attributed to difference in processing method as the olive cake available in India is crude cake and was not subjected to solvent extraction, which explains the high EE% of the analyzed samples. CP content also varies according to type of olive cake like stoned olive cake has lower CP than partly stoned olive cake. The moisture content of olive cake in this study was considerably lower than the previous reports by Al-Jassim *et al.*, [13], Al-Masri, [24], Martín García *et al.*, [26], Al-Masri, [31] and Molina- Alcaide *et al*. [35], which may be due to the fact that olive cake available for this study was heaped outside the processing mill and was exposed to air and sunlight causing appreciable level of drying before the sample was collected. Besides the fruits were harvested after complete ripening which eventually contributes to low moisture content of olive cake. However similar moisture content has been reported by Abbeddou *et al*. [36].

**Table 6:** *In vitro* degradability variable of complete feed comprising of ADF replacement at variable levels by lime treated olive cake

Levels	IVDMD (%)	TDOM (mg/200 mg DM)	Gas production (ml/200mgDM)	MBP (mg/200 mg DM)	EMP (%TDOM)	PF
Level 0	51.25±1.25	102.94^bc^±0.80	30.00^c^±0.41	36.94^abc^±1.28	35.87^abc^±1.07	3.43^ab^±0.06
Level 1	48.75±1.25	100.00^ab^±0.74	30.25^c^±0.48	33.45^a^±1.66	33.42^a^±1.44	3.31^a^±0.07
Level 2	51.25±1.25	103.25^c^±0.72	29.75^c^±0.63	37.80^abcd^±1.70	36.59^abc^±1.52	3.48^ab^±0.08
Level 3	52.50±1.44	105.00^c^±0.61	31.25^c^±0.75	36.25^ab^±1.20	34.54^ab^±1.28	3.36^ab^±0.07
Level 4	55.00±3.54	112.00^e^±1.02	33.00^d^±0.71	39.40^bcde^±2.49	35.13^abc^±1.92	3.40^ab^±0.10
Level 5	56.25±1.25	111.00^de^±1.17	31.25^c^±0.48	42.25^cde^±0.91	38.06^bc^±0.71	3.55^ab^±0.04
Level 6	56.25±2.39	109.00^de^±0.98	30.00^c^±0.41	43.00^def^±1.02	39.44^c^±0.78	3.63^b^±0.05
Level 7#	55.00±2.04	108.75^d^±1.42	27.25^b^±0.48	48.80^g^±2.25	44.82^d^±1.52	4.00^c^±0.11
Level 8	51.25±2.39	102.75^bc^±1.36	25.00^a^±0.41	47.75^fg^±2.25	46.41^d^±1.58	4.12^c^±0.12
Level 9	48.75±3.75	97.50^a^±1.10	24.00^a^±0.41	44.70^efg^±1.60	45.82^d^±1.22	4.07^c^±0.10

*IVDMD=*In vitro* dry matter degradability, TDOM=Truly degradable organic matter, MBP=Microbial biomass production, EMP=Efficiency of microbial biomass production, PF=Partitioning factor,

**abcdefg:** Mean values bearing different superscripts within a column differ significantly (p<0.01),

# Level 7 showed comparatively higher values

The NDF and ADF content of the crude olive cake were found to be 80.33 and 62.00%, respectively on DMB. The values were higher to those recorded by Ahmad [4], Ashraf [5], Bashir [6], Al-Jassim *et al*. [13], Martín García *et al*. [26], Molina-Alcaide *et al*. [27], Chiofalo *et al*. [29] and Rowghani *et al*. [37]. However, values were similar to those found by Ohlade-Becker [11] Abarghoei *et al*. [38] and Al-Masri [31]. The variations recorded in fiber composition in the olive cake may be due to different geographical location and processing methods. The increased cell wall constituents may also be because of the fact that the olive cake we used was stoned rather than partly stoned olive cake. The NDF and ADF were found higher than reported by Ahmad [4], Ashraf [5] and Bashir [6], which may be attributed to different year and origin of olive fruit in spite of same place and method of extraction. The hemicellulose content was similar as reported by Tufarelli *et al*. [39].

Slaked lime treatment was used as per Ashraf [5] to improve the IVDMD values of crude olive cake and to alleviate the digestibility depression casted by high-fat content. There was a significant decrease in OM, NDF, ADF and hemicellulose percentage in lime treated olive cake. Similar decrease has been reported by Abo Omar *et al*. [40]. However, there was a significant increase in total ash and calcium content owing to lime treatment.

Olive cake as a feedstuff blurs the demarcating criteria between roughage and concentrates. By standard classifying pattern of feedstuffs, it should be classified as extremely poor quality roughage. However, its higher EE% makes it better concentrate. Although, it is an oil seed cake, it is extremely poor in protein and in contrast, it is almost 50-60% fiber, which makes it an ideal candidate for quality improvement through lime treatment.

### Nitrogen fractionation of crude olive cake

The crude olive cake was evaluated for protein fractions as per CNCP system by Sniffen *et al* [17]. It was found that 33.69% of protein is instantly degradable which is represented by Fraction A. Fraction B_2_ which is slowly degraded amounts to 5.14%. Fraction C which is undegradable was found to be 21.71%. Many previous workers have reported protein degradability of olive cake is poor, owing to the fact that 75-90% of the nitrogen is linked to the ligno-cellulose fraction [26-27] thereby resulting in low nitrogen solubility [9]. However, as per our results, the ADIN content is limited to about 22% of total nitrogen, which translates into 78% degradable and thus available fraction. This was in accordance to the interpretations of Ashraf [5] and Bashir [6], which was based on the low fecal nitrogen excretion found in their studies. Interestingly, both these studies were conducted at the laboratory of the author of this study and are pilot study on olive cake feeding in India. From our results we inferred that Indian olive is different in composition from European or Mediterranean olive as far as level of lignin bound nitrogen is concerned and thus can be classified as average contributor of CP to ration and this makes it quite iso-nitrogenous to maize and other common feed grains. However keeping in view the limitations of the trial including the processing of olive cake at extraction mill, the study needs to be researched more.

### *In vitro* DM degradability variables

Ten isonitrogenous complete diets containing variable levels of lime treated olive cake on ADF replacement basis were formulated and tested through *in vitro* gas production technique as per Menke and Steingass [19].

**Figure-1:**
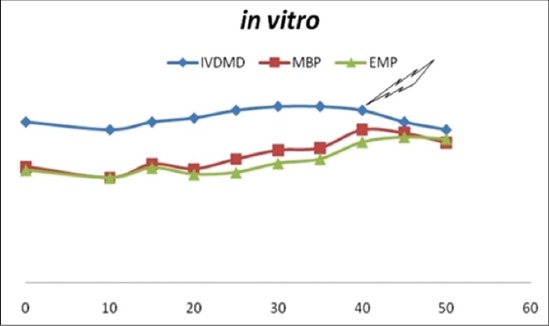
*In vitro* dry matter degradability, microbial biomass production, efficiency of microbial biomass production

The IVDMD values obtained were compared to select the complete feed with maximum inclusion level of lime treated olive without significant decrease in IVDMD. The IVDMD values obtained were intermediate to the values reported by Vera *et al*. [8] and Shabtay *et al*. [41] but were similar to that reported by Brozzoli *et al*. [42]. The IVDMD values for all inclusions levels were comparable (p>0.05) however, point of inflection was observed at 40% inclusion as shown in Figure-1. The highest TDOM was observed at 25% ADF replacement but was comparable with levels 30% and 35%. The 40% ADF replacement was comparable with 30% and 35% levels. The MBP value was highest in 40% replacement level and differed significantly (p<0.001) from all the lower levels. EMP and PF value were highest in 45% ADF replacement level but were comparable with levels 40% and 50% levels. It appears that lime treatment was able to improve ADF utilization, while concurrently alleviating the depressing effect of fat over digestibility. Alleviation of digestibility depression by fat may be attributed to formation of calcium salts of fatty acids by lime treatment [5].

## Conclusion

In our study, we concluded that there is a comparable difference in composition of Indian olive cake when compared to European olive cake. The most important finding was that about 78% of nitrogen present in Indian olive cake is available to the animal. We concluded from *in vitro* analysis that olive cake can be included in complete feed at 30% level (w/w; 40% ADF replacement) for feeding in small ruminants without compromising *in vitro* degradability.

## Author’s Contribution

AI carried out the research work and drafted the manuscript, RKS and AR planned and supervised the research work, BAM and JF helped in conducting lab trial and AI and AR revised the manuscript. All authors read and approved the final manuscript.
